# Comparative Review of Malignant Melanoma and Histologically Well-Differentiated Melanocytic Neoplasm in the Oral Cavity of Dogs

**DOI:** 10.3390/vetsci8110261

**Published:** 2021-11-02

**Authors:** Won Suk Kim, Arathi Vinayak, Barbara Powers

**Affiliations:** 1 Department of Surgical Oncology, VCA West Coast Specialty and Emergency Animal Hospital, 18300 Euclid Street, Fountain Valley, CA 92708, USA; arathi.vinayak@vca.com; 2 Antech Diagnostics, 17620 Mt Hermann St, Fountain Valley, CA 92708, USA; barbara.powers@antechmail.com

**Keywords:** oral tumor, melanoma, melanocytic neoplasm, mitotic index

## Abstract

Oral malignant melanoma (OMM) is the most common neoplasm of the canine oral cavity. It is characterized by its aggressive local disease as well as its high rate of lymphatic invasion and distant metastasis. OMM carries a poor prognosis, with most patients succumbing to the disease due to progression of the neoplasm. Histopathologically, OMM is characterized by significant nuclear atypia, a mitotic index of greater than 4/10 hpf, and evidence of vascular invasion or metastasis. Clinically, these lesions can become locally invasive, causing lysis of bones and severe inflammation of the surrounding soft tissue. With time, these lesions can spread to the regional lymph node and to the lungs and other organs. Prognosis can vary depending on the size of the primary tumor, regional node involvement, and distant metastatic disease; however, multiple studies report a relatively short median survival time ranging from less than 4 months to 8 months. Histologically well- differentiated melanocytic neoplasms (HWDMN) are a variant of OMM and sometimes referred to as canine oral melanocytic neoplasms of low malignant potential. Unlike OMM, patients with HWDMN have longer survival times. Histopathologically, HWDMNs have well-differentiated melanocytes with a low mitotic index of 3 or less per 10 hpf and minimal nuclear atypia. HWDMNs have better prognosis with a mean survival time of up to 34 months. This article is a comparative review of OMM and its less aggressive counterpart.

## 1. Introduction

Canine oral malignant melanoma (OMM) is an aggressive cancer of the oral cavity in dogs that accounts for 11.5% [1] to 17.1% [2] of all oral tumors and is the most common malignant oral tumor, making up between 33% [3]–35.8% [1] of all malignant oral tumors. This devastating cancer is characterized by its marked local destruction as well as a high propensity to metastasize [3,4,5,6]. Prognosis with OMM is poor, with a reported median survival time (MST) of 65 days without treatment [7]. The primary treatment of OMM is wide-margin surgical resection [8] with adjuvant treatments such as intensity-modulated radiation therapy (IMRT), considered if margins are narrow/incomplete, or coarse fraction radiation, considered as the sole treatment from a palliative standpoint [9,10]. The staging of OMM is based on the World Health Organization (WHO) staging scheme and is dependent on the tumor size and regional and distant metastatic disease [11]. The overall reported MST following surgical excision vary depending on the stage of disease; Stage I, II, and III disease carry MSTs of 17–18 months, 5–6 months, and 3 months, respectively [12,13]. The survival times following palliative radiation therapy (RT) also varies from 5.3 to 11.9 months [10,14,15,16,17], with a longer survival time of 11.9 months reported with concurrent cisplatin or carboplatin administration along with radiation therapy [17]. Systemic treatments such as immunotherapy and chemotherapy have been studied; chemotherapy for OMM has not been promising and immunotherapy, mainly the Oncept^®^ vaccine, shows varying results [8,9,18,19]. With limited treatment options and short survival times, OMM truly is a devastating cancer. A retrospective study reported that 32% of dogs with melanocytic neoplasm were alive at follow-up or died of causes unrelated to the neoplasm and concluded that not all melanocytic oral neoplasm should be considered highly malignant [20]. A variant of OMM, known as histologically well-differentiated melanocytic neoplasm (HWDMN), with a low mitotic index (MI < 4 per 10 hpf), has a more favorable prognosis compared to traditional OMM [21]. Patients with HWDMN have been reported to have prolonged MST with local surgical excision only [21]. An MST of 34 months has been reported in patients with HWDMN in the literature, with a low recurrence rate of 3.1% [21]. Two other variants of OMM, balloon cell melanoma and signet ring melanoma, have been reported in two histopathologic studies; however, the clinical ramifications are unknown, as survival times were not reported [22,23]. The purpose of this review is to shed light on the lesser-known variant and to provide better understanding of this disease compared to the more common aggressive variant of malignant melanoma in the oral cavity of dogs.

## 2. Signalment and Presenting Signs

OMM is reported to be a disease that affects geriatric dogs. The mean age of those affected varies between 9–11.4 years of age [13,24], depending on the source, and can be seen in dogs from 1–17 years of age. Similarly, HWDMN has been reported in dogs between 3–17 years of age with a mean of 10.5 years [21]. 

There is contradicting information regarding gender prevalence for OMM. Ramos-Vara reported no difference in OMM prevalence between the genders, while other studies indicate a higher prevalence in male dogs [24,25,26], similar to human literature where males are more affected compared to females, with male to female ratios of up to 2–1 reported in some cases [27,28,29,30]. Certain breeds have been noted to be predisposed to developing OMM. Cocker Spaniels, Poodles, Pekinese, Gordon Setter, Chow Chow, Golden Retrievers, mixed-breed dogs, and Dachshunds are reported to be the most common breeds affected by OMM [24,31]. HWDMNs have similarly been reported in Golden Retrievers, Labrador Retrievers, Doberman Pinscher, Irish Setters, Cocker Spaniels, Beagles, and mixed-breed dogs [21].

**Figure 1:**
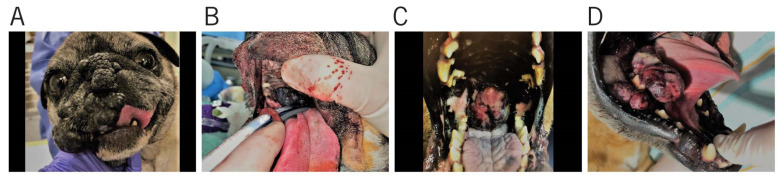
Similar clinical presentation of histologically confirmed histologically well-differentiated melanocytic neoplasm (HWDMN) and oral malignant melanoma (OMM): (**A**) Right rostral muzzle and nasal planum pigmented HDWMN obliterating normal anatomy; (**B**) Pigmented HWDMN of the hard palate extending into the soft palate; (**C**) Variably pigmented proliferative OMM of the soft palate; (**D**) Variably pigmented proliferative OMM of the right caudal mandible.

Presenting clinical signs of canine OMM can include bleeding, ptyalism, dysphagia, halitosis, and occasionally, mandibular fracture [3,4,32]. OMM can appear as multiple small brown-to-black masses, but also may be a large, irregular or flat mass, with varying level of pigmentation [33]. Similarly, dogs with HWDMN may present with pigmented, discrete to large lesions on the lips and within the oral cavity (Figure 1). Interestingly, in humans, OMM was reported in individuals of varying ages ranging from 22–83 years of age and a mean age of 56 years [34]. The incidence of prepubertal children with OMM is exceedingly uncommon [35]. The ethnic groups most affected by OMM are Japanese, black Africans, Native Americans, and Hispanics [36]. 

## 3. Gross Appearance and Cytology

Grossly, OMM can vary in its appearance, with colors ranging from gray to red, and even dark blue [3]. Pigmentation does not provide a diagnosis of melanoma, as other neoplastic and nonneoplastic lesions can appear similar. The presence of melanin within the tumor can indicate melanoma; however, approximately one third of all melanoma cases in dogs are amelanotic malignant melanomas [13,24]. Furthermore, amelanotic malignant melanomas may grossly resemble sarcomas, lymphomas, carcinomas, and osteogenic tumors [3]. 

An initial diagnosis of melanoma can be achieved by cytologic assessment of the lesion as a screening measure, followed by histopathologic evaluation [3]. Cytologically, malignant melanoma has significant anisocytosis and anisokaryosis, hyperchromasia, abnormal chromatin clumping, one or more nucleoli, and atypical mitotic figures. These cells can be typed as epithelioid, round, spindle, or a mixture of these [3,20]. OMM cannot be differentiated from HWDMN based on cytology alone, as the cell population and appearance are similar. It can also be difficult to obtain a definitive diagnosis in cases of amelanotic and poorly differentiated melanomas, and immunohistochemistry using monoclonal and polyclonal antibodies is commonly employed to obtain a definitive diagnosis [3].

## 4. Staging Diagnostics

For dogs with OMM, the size of the primary tumor is reported to be prognostic [12]. Staging of canine OMM is based on the WHO TNM grading scheme (Table 1). Stage I diseases are those with tumors <2 cm diameter, stage II tumors are 2 cm to <4cm diameter, and stage III tumors are 4 cm or greater and/or metastasized to the regional lymph node. Those with distant metastatic disease are considered stage IV. A similar staging system has not been reported for HWDMN. 

A minimum database for staging includes a detailed history, physical exam, baseline bloodwork, urinalysis, chest radiographs, abdominal ultrasound, and local lymph node aspiration [12]. While HWDMN has a lower metastatic potential, staging diagnostics remain largely the same. All four lymph centers (ipsilateral and contralateral mandibular and medial retropharyngeal) should be aspirated for OMM. It is well known that lymph node size is not a reliable gauge of metastasis [37,38] and fine needle aspirate is reported to have a 100% sensitivity and 96% specificity when detecting lymphatic metastasis of solid tumors [38]. In addition, it was reported that 33% of patients with OMM had lymphatic metastasis to all four lymph centers, and 42% of patients had metastasis to the contralateral side [39]. Computed tomography (CT) imaging of the head and chest can be performed for staging and surgery planning [6]. 

## 5. Histopathology

As stated previously, the staging of canine OMM traditionally has been based on the WHO TMN classification system [11], which does not take into account the mitotic index (MI). MI refers to the percentage of cells in a population that are actively undergoing mitosis. A study by Hanh et al. noted that the WHO classification did not meet the objectives of tumor staging, which is to determine the extent of disease and to aid in recommending an effective treatment plan [40]. Their proposed alternative staging system was based on location, size, and the mitotic index of tumors, with better prognostic predictability. Their proposition was further corroborated by a recent study that found that large and non-pigmented tumors, necrosis, ulceration, high MI, and p53 expression are poor prognostic indicators [3]. Mitotic index is one of the most important criteria in differentiating HWDMN from OMM.

**Figure 2:**
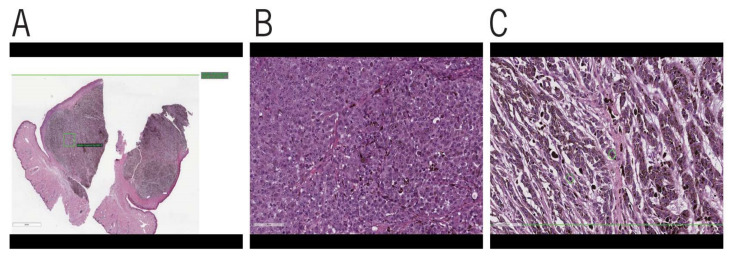
Histopathologic features of OMM: (**A**) Low magnification, bar = 4 mm. Lightly pigmented mass beneath mucous membrane. Mitotic count of 9 per 10 high powered fields (2.37 mm^2^). The area framed by the green lines denotes the area assessed by the artificial intelligence (AI) software used by Antech diagnostics to calculate mitotic index (MI) in addition to manual counts done by the pathologists in the same field as a cross-check. The green circles indicate a location where mitotic figures were noted by the AI system. (**B**) Moderate magnification, bar = 90 um. Poorly pigmented tumor cells with moderate to marked nuclear atypia, large prominent nucleoli, and numerous mitotic figures. (**C**) High magnification, bar = 70 um. Lightly pigmented tumor cells with mildly pleomorphic oval nuclei and prominent nucleoli. Green circle indicates a location where mitotic figures were found by the AI software used by Antech diagnostics to calculate MI.

Histopathologically, OMM can be composed of cell types ranging from epithelioid, spindle, mixed, dendritic, and signet, with epithelioid cells reported to be the most common in one study [20] (Figure 2). A different study reported mixed cell types as the most common [24]. An important feature of OMM is junctional or intraepithelial tumor growth. It is common to see single atypical cells or cell nests with infiltration of the surface epithelial basal layer with anaplasia, varying levels of mitotic activity, and cell pleomorphism. In addition, invasion into the lymphatics and small veins is frequently seen [41]. The cells in the nests may be heavily pigmented, but there may be variations in the degree of pigmentation, with some that have no visible pigment [42]. It is reported that a third of canine melanomas are amelanotic [13]. 

**Figure 3:**
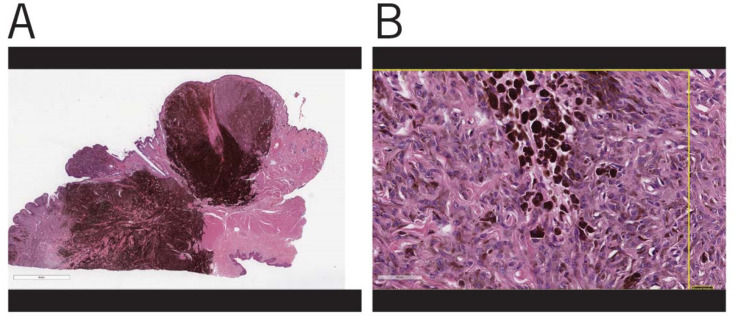
Histopathologic features of HWDMN: (**A**) Heavily pigmented pedunculated mass at mucocutaneous junction with early submucosal extension. Bar = 4 mm; (**B**) Areas of heavily pigmented tumor cells adjacent to less pigmented tumor cells. Nuclei are mildly pleomorphic, oval and have prominent nucleoli, but no mitotic figures. The area framed by the yellow lines denotes the area assessed by the artificial intelligence (AI) software used by Antech diagnostics to calculate MI in addition to manual counts done by the pathologists in the same field as a cross-check. Bar = 60 um.

In contrast, in HWDMNs, the neoplastic cells appear uniform in size and are either round or elongated. The nuclei in these cells are small and have a round to elongated appearance. The mitotic index of these neoplasms was reported to be low, with less than 4 per 10 hpf [21] (Figure 3). 

Immunohistochemistry (IHC) is the gold standard for the diagnosis of tumors of melanocytic derivation if a diagnosis is not possible on a standard hematoxylin and eosin (H&E) prep. The distinguishing characteristic between an OMM and HWDMN is the low mitotic index of less than 4 per 10hpf and the well-differentiated appearance of the cells [21]. Thus, an examination of the cytomorphologic appearance in addition to IHC analysis may be important. It is imperative that clinicians are vigilant in their review of the microscopic findings in histopathology reports to determine whether HWDMN is a possible differential based on the differentiation of the cells and MI. Clinicians should be aware that the histopathologic diagnosis for both OMM and HWDMN will be malignant melanoma. Differentiating between the two diagnoses drastically changes prognosis with treatment for the patient and may affect an owner’s decision on whether or not to pursue treatment.

With regards to IHCs, most melanomas, including HWDMN, are cytokeratin negative and vimentin positive [3], but sarcomas can have similar staining patterns [43]. Other monoclonal antibodies (mAb) are available to aid in the detection of melanoma in humans and dogs [44]. Markers, including vimentin, S-100 protein, neuron-specific enolase (NSE), Melan A, PNL2, human melanosome-specific antigens -1 (HMSA-1), and HMSA-5 have been assessed for their potential in aiding the diagnosis of canine malignant melanoma. 

In a study by Ramos et al. it was reported that 100% of melanomas were positive for vimentin. The authors concluded that although vimentin is a sensitive marker for mesenchymal cells, due to its low specificity, it should considered a preliminary screening tool [24]. S-100 protein is an intracellular and intranuclear acidic, calcium-binding protein [45], commonly found in melanocytic lesion. However, this protein is also found in other nonmelanocytic cells, including Langerhans cells, macrophages, chondrocytes, neurons, and others [24,46]. Approximately 76–84% of canine melanoma tumors were positive for S-100 protein [24,47]. NSE is found in peripheral and central neuroendocrine cells, neurons, and melanocytes, and tumors derived from these cells [46]. Although this protein is not very specific for melanomas, 89.1% of melanomas were positive for this protein in one study [24]. Positive immunoreactivity for vimentin, S-100 and NSE along with a negative cytokeratin immunoreactivity supports a melanoma diagnosis [44]. Melan A is a protein that elicits a cytotoxic T-cell response and is considered the most specific IHC test used due to its narrow tissue distribution [3]. Ramos-Vara et al. reported that of the 122 canine oral melanomas and 7 metastatic melanomas, 92% were Melan A positive, 100% vimentin positive, 76% S100 positive, and 89.1% NSE positive [24]. As 32% of the total number of tumors were nonpigmented; the assumption is that a significant proportion of amelanotic melanocyte tumors were reactive to Melan A. In the same study, a large population of non-melanocytic tumors (not amelanotic melanomas) were tested for Melan A, with only four tumors reacting to the protein. These findings led to the conclusion that Melan A is a sensitive and specific marker for melanocytic neoplasms [24]. PNL2 is a monoclonal antibody that targets fixative resistant melanocytic antigen and was developed for human melanoma diagnosis [48]. The antigen targeted by PNL2 is reported to be detectable even after melanin bleaching and decalcification, adding to PNL2′s value in diagnosing malignant melanoma [48]. Furthermore, PNL2 has a higher sensitivity compared to Melan-A and lacks cross-reactivity with nonmelanocytic neoplasms [49]. PNL2 is a reliable marker in the identification of canine melanomas, with close to 100% sensitivity when used together with Melan A and tyrosinase [49].

In human medicine, the development of murine antibodies targeting melanoma-associated antigens, such as melanosome [50], has further solidified the value of IHC in diagnosing melanoma [3]. mAb used in human medicine for diagnosis of melanomas that cross-react with canine melanomas include human melanosome-specific antigens-1 (HMSA-1), HMSA-5, and Melan A [44,50]. HMSA-1 and HMSA-5 individually are reported to react with 60% and 69% of canine melanomas, respectively. However, when used together, they react with 83% of canine melanomas [3].

IBF9 is the only known murine mAb generated specifically for canine melanoma detection [3,51]. IBF reacted positively to only 63% of melanomas tested and reacted to 30% of non-melanocytic tumors such as cutaneous lymphoma and basal cell tumors [51]. IBF9 can cross-react with other tumors, but additional antibody IHC testing and cytomorphologic examination can make IBF9 more useful [3]. Similarly, using multiple antibodies can help to increase diagnostic accuracy. A cost-effective and efficient immunodiagnostic cocktail, containing PLN2, Melan-A, TRP-1, and TRP-2, has been found to be effective, with 93.9% sensitivity and 100% specificity in canine amelanotic melanocytic lesions [52]. 

A study in humans assessed the immunohistochemical expression of different tumor-suppressive genes including, p53, p16, Rb, and pRb2/p130 proteins. This study revealed that p53 expression was associated with poor prognosis, while the expression of pRb/p130 proteins were associated with a higher survival rate [53]. To the author’s knowledge thus far, evaluation of these suppressive genes has not yet been assessed in veterinary medicine but could be of great value in the treatment of OMM in veterinary patients, as it may help elucidate the subset of patients that may fare better with treatment. Further studies examining the correlation between these genes and stage of OMM in veterinary patients is required.

## 6. Treatment

Surgery is the most effective treatment for the elimination of the primary tumor [8]. Prior to surgery, computed tomography (CT) is commonly utilized for the staging of OMM and treatment planning [6]. Because of OMM’s propensity to metastasize, systemic treatment should ideally be a consideration. However, despite this recommendation, no significant improvement in survival times have been noted in multiple protocols that have been investigated [8,13,18,54]. Tuohy et al. found that surgery with adjuvant therapy did not improve the survival times in patients with OMM [8]. Rather, patients that received adjuvant therapy had a shorter mean survival time (MST) of 396 days compared to 874 days for dogs that had surgery only. The adjuvant therapies for this study included different combinations of maximum tolerated chemotherapy, metronomic chemotherapy, radiation therapy, and a melanoma vaccine. In a study by Boston et al., patients treated with surgery alone versus surgery followed by systemic adjuvant, had an MST of 355 days and 352 days, respectively [18]. Adjuvant therapies in this study included hypofractionated radiation therapy, chemotherapy, metronomic chemotherapy, and melanoma vaccination. In the case of HWDMNs, however, Esplin noted that patients treated only with local excision had an MST of 34 months, with extremely low recurrence rates and low metastatic potential—with only 2 of the 64 dogs having suspected metastasis [21]. Thus, wide-margin surgery may be sufficient for HWDMNs as the sole treatment, due to a low recurrence rate and low potential for metastasis.

Radiation therapy (RT) may be useful for the control of local disease in certain cases. A few studies have noted that chemotherapeutics in conjunction with radiation therapy (not surgery) was successful in prolonging the median time to progression and survival time [17,55]. Historically thought of as radio-insensitive, many studies now suggest radiation therapy as a reasonable option for OMM, especially if the tumors are smaller. Proulx et al. found that dogs without bony destruction had longer disease-free intervals and overall survival compared to those with bone invasion after RT [14]. The response rates to RT ranged from 82–94% and an MST of 5.3–11.9 months [14,16,17,33,55,56], with the longer survival times reported with concurrent carboplatin or cisplatin administration in some studies [17]. In a study by Cancedda et al., the authors noted increased time to progression, with median of 205 days for those that received RT and temozolomide, compared to 110 days for those that received RT only, though survival times were not significantly different [55]. It is possible that a subset of OMM that have responded well to radiation therapy and surgery were dogs with HWDMN, as the mitotic index in most of the current published studies was not assessed. To the authors’ knowledge, efficacy and outcome information with RT in dogs with HWDMNs has not been reported. 

Efficacy of numerous chemotherapeutics has been studied in both human and veterinary medicine. Treatments with carboplatin, cisplatin, and melphalan have been assessed in multiple veterinary studies, but have not shown improvement in the overall survival time and showed low response rates. Multiple studies evaluating different chemotherapies as the sole treatment [57,58] and as an adjuvant treatment following surgery [54,59] or radiation therapy [14,16] report underwhelming results. Similarly, in humans, the role of chemotherapy remains unclear, with multiple studies reporting response rates as low as 10% and a general lack of efficacy in this disease [60,61,62,63,64]. 

Immunotherapy for the treatment of canine OMM is an area of active research and development. The goal of immunotherapy is to activate the immune system to modulate the progression and spread of melanoma. A variety of biological agents have been examined for their potential to elicit an antitumor response by the immune system. Bacillus Calmette–Guerin (BCG), IFN-alpha, IL-2m and immunization activators have shown promise [65]. A xenogenic DNA-encoding tyrosinase protein vaccine, the Oncept^®^ vaccine, has been available since 2007, and has been reported to be well-tolerated, with minimal side effects [9,19,66]. While initial prospective studies showed improvements in survival times when used as an adjuvant [19,66], the more recent retrospective studies have reported equivocal benefits of the melanoma vaccine in terms of the progression-free survival, disease-free interval, and MST compared to patients who did not receive the vaccine in an adjuvant manner [8,18,67].

In humans, an area of intense research is the development of therapies to improve immune-mediated destruction of neoplastic cells [13]. T-cell mediated cytotoxicity was found to be impaired when T-cell programmed-death-receptor-1(PD-1) and programmed death receptor ligand (PD-L1), expressed by antigen-presenting cells and cancer cells, interact and lead to downregulation of T-cell activation. Melanoma has been shown to express PD-L1 on the cell surface [68]. This disruption in immunity allows for the tumor to evade the immune system and continue to grow [64]. One strategy that is under investigation is the inhibition of PD-L1 and PD-1 interaction, thereby keeping the T-cell mediated response intact. One example of this is the pembrolizumab, which is an antibody against PD-1. The drug underwent phase 1 trial, with promising results in 2014; 88% of responses to the drug were ongoing at median follow-up of 8 months [68]. This is consistent with previous findings where immunotherapies were noted to have long-lasting clinical benefit [69,70]. Pembrolizumab was approved for treatment of recurrent or metastatic cervical cancer by the Food and Drug Administration on 13 October 2021. These results provide some hope for systemic treatments of OMM. Research into the feasibility of this therapy in veterinary patients is needed.

## 7. Prognosis

Dogs with OMM historically have poor prognosis, with untreated oral melanoma patients having an MST of 65 days [7]. MSTs for stages I, II, and III are 17–18 months, 5–6 months, and 3 months following surgery, respectively [71]. As stated previously, a melanoma vaccine and RT with concurrent chemotherapy may improve survival times based on some studies. In contrast, patients with HWDMN have a prolonged MST of 34 months and a recurrence rate of 3.1%.

## 8. Conclusions

There is a lot of information in the veterinary literature regarding OMM. The one commonality amongst the studies is that OMM is a devastating disease with aggressive local disease and high propensity for spread. Prognosis is poor, with most patients succumbing to disease progression even in the face of aggressive surgery, and with adjuvant treatments having questionable efficacy in improving outcome. However, a variant of OMM known as HWDMN has a different biological behavior that affords patients longer survival times. As such, it is important to recognize that despite OMM diagnosis, a review of the histopathologic characteristic of the tumor is crucial, as it may yield a more favorable prognosis. The authors strongly recommend reviewing the pathologist’s findings and comments to ensure HWDMN is not missed, as this diagnosis could provide a more accurate prognosis and determine a proper treatment plan.

## Figures and Tables

**Table 1:** Adapted from World Health Organization (WHO) primary tumor, regional lymph node, metastasis (TNM) staging scheme for dogs with oral malignant melanoma (OMM) [11].

**T: Primary Tumor**
T0—No evidence of tumor
T1—Tumor < 2 cm maximum diameter
T2—Tumor 2–4 cm maximum diameter
T3—Tumor > 4 cm maximum diameter
**N: regional Lymph node**
N0—No evidence of regional involvement
N1—Movable ipsilateral nodes
N2—Movable contralateral or bilateral nodes
N3—Fixed nodes
(A) Nodes do not contain tumor
(B) Nodes contain tumor
M: distant metastasis
M0—No evidence of distant metastasis
M1—Distant metastasis (including distant nodes)
**Stage grouping**
Stage I—T1 N0 M0
Stage II—T2 N0 M0
Stage III—T2 N1 M0 or T3 N0 M0
Stage IV—Any T, any N and M1

Note. Adapted from TNM Classification of Tumours in Domestic Animals (1 st ed., p 23) by L.N. Owen, 1980, WHO.

## Data Availability

Not applicable.

## Abbreviations

OMM	Oral Malignant Melanoma
HWDMN	Histologically Well-Differentiated Melanocytic Neoplasm
MST	Median Survival Time
IMRT	Intensity-Modulated Radiation Therapy
RT	Radiation Therapy
WHO	World Health Organization
